# Clinical experience and perception of risk affect the acceptance and trust of using AI in medicine

**DOI:** 10.3389/fdgth.2025.1620127

**Published:** 2025-09-02

**Authors:** Peter J. Schulz, Kalya M. Kee, May O. Lwin, Wilson W. Goh, Kendrick Y. Chia, Max F. K. Cheung, Thomas Y. T. Lam, Joseph J. Y. Sung

**Affiliations:** ^1^ Wee Kim Wee School of Communication, Nanyang Technological University, Singapore, Singapore; ^2^ Lee Kong Chian School of Medicine, Nanyang Technological University, Singapore, Singapore; ^3^ Center of AI in Medicine, Nanyang Technological University, Singapore, Singapore; ^4^ Nethersole School of Nursing, The Chinese University of Hong Kong, Sha Tin, Hong Kong

**Keywords:** artificial intelligence, colonic polyps, colonoscopy, behavior, acceptance

## Abstract

**Background & aims:**

As Artificial Intelligence (AI) is progressively making inroads into clinical practice, questions have arisen as to whether acceptance of AI is skewed towards certain medical practitioner segments, even within particular specializations. This study aimed to examine distinct AI attitudes (including trust and acceptance) and intended behaviors among clinicians from contrasting backgrounds and levels of seniority/experience when interacting with AI.

**Methods:**

Based on the results we divided participants into four groups, those who have (i) low experience and low risk perception, (ii) low experience and high risk perception, (iii) high experience and low risk perception, and (iv) high experience and perceived risk of AI use to be high. An ANCOVA model was constructed to test whether the four groups differ regarding their overall acceptance of AI.

**Results:**

Data from 319 gastroenterologists show the presence of four distinct clusters of clinicians based upon experience levels and perceived risk typologies. Analysis of cluster-based responses further revealed that acceptance of AI was not uniform. Our findings showed that clinician experience and risk perspective have an interactive role in influencing AI acceptance. Senior clinicians with low-risk perception were highly accepting of AI, but those with high-risk perception of AI were substantially less accepting. In contrast, junior clinicians were more inclined to embrace AI when they perceived high risk, yet they hesitated to adopt AI when the perceived risk was minimal.

**Conclusions:**

More experienced clinicians were more likely to embrace AI compared to their junior counterparts, particularly when they perceived the risk as low.

## 1 Introduction

Artificial Intelligence (AI) is making inroads into clinical practice. Its applications in healthcare are wide and varied, encompassing diagnosis, risk assessments, disease management, complication surveillance, and prediction of outcome (1). AI is set to disrupt the conventional workflow of healthcare systems while creating new opportunities for healthcare (2).

AI is anticipated to be more extensively used in specialties that utilize medical imaging heavily, such as radiology, pathology, and gastroenterology (3). The application of AI in the specialty of radiology has been extensively studied. However, to our knowledge, research is the specialty of gastroenterology is limited. Although these domains utilize medical imaging, the AI applications in the domains are diverse (4). In gastroenterology, this includes risk stratification scoring systems, predicting treatment responses, and evaluating the quality of endoscopic examinations (4, 5). Recently, AI has been applied in invasive and risky therapeutic procedures such as assessing adequacy of biopsy sample endoscopic ultrasounds guided fine-needle aspiration (6, 7). The increasing incorporation of AI in clinical practice raises questions regarding clinicians' trust and acceptance of AI technologies in clinical decision-making process and guiding therapies.

Trust and acceptance of AI in healthcare are influenced by clinicians' general expectations of AI and their overall attitudes towards this rapidly evolving technology. Gastroenterologists generally believe that AI will impact the field positively (8). However, concerns remain regarding specific uses of AI. In our previous study, we found a direct correlation between AI acceptance and willingness to trust and use the technology (9). However, levels of trust and acceptance varied with the invasiveness of therapeutic procedures. These findings highlight the importance of fostering clinician acceptance and trust to increase willingness to integrate AI into medical practice.

Many factors can impact trust and acceptance of AI among clinicians, such as the support from senior clinicians (10). However, there is some hesitancy to encourage junior clinicians to use AI. Senior clinicians believe that junior clinicians may become overly reliant on AI, and subsequently lack critical thinking skills (10). Another issue is of perceived practical value. Senior clinicians, with ample experience, were found to benefit less from AI than their junior colleagues (11). Another study reported that senior clinicians, specifically senior consultants and consultants, view the implementation of AI as less risky compared to residents and fellows (9). However, few studies have examined the associations between levels of experience and clinician perceptions towards AI in healthcare (12).

A research gap exists in understanding how clinicians with varying levels of experience perceive dissimilar risk types of AI applications. The key research question for this study was: How does level of experience impact attitudes towards AI use for clinical practice? Hence this study aimed to examine AI attitudes and intended behaviors among clinicians from differing experience levels when interacting with AI.

## 2 Methods

### 2.1 Design

This was a cross-sectional study among gastroenterologists from Asia-Pacific countries/regions (Australia, Brunei, China, Hong Kong, India, Indonesia, Japan, Singapore, South Korea, Taiwan). Eligible participants were contacted via email. Clinicians were eligible if they were (i) practicing clinicians in the field of gastroenterology or gastrointestinal surgery, (ii) practicing in the Asia-Pacific region, and (iii) trained in performing colonoscopy for colorectal cancer screening. Ethical approval was obtained from the university institutional review board (IRB-2022-756).

### 2.2 Questionnaire and measures

The online questionnaire included questions about participants' socio-demographic characteristics, work-related details, perceptions, trust and acceptance of Artificial Intelligence (AI). The questionnaire was developed based on established and validated frameworks, namely, the expectancy-value framework, and major constructs of the Theory of Planned Behaviour research framework (13, 14), and the Technology Acceptance Model measures (9, 15, 16). Items used to measure trust and acceptance of AI in healthcare, and risk tolerance were adapted from prior studies (17, 18). To measure the trust and acceptance of specific AI applications, three scenarios covering the detection of polyps in the colon and rectum, characterization of the pathological nature of these polyps, and removal of polyps (polypectomy) plus treatment of resulting complications from polypectomy were included. Scenarios were designed to encompass increasing levels of sophistication (in AI applications).

In line with the theoretical foundations, some key psychological constructs such as attitude, perceived behavioral control, and perceived usefulness were included to understand individual variation in AI acceptance. Attitude, derived from the Theory of Planned Behaviour, reflects a participant's perception of AI use in clinical decision-making (e.g., whether it is perceived as beneficial, appropriate, or trustworthy). By including these variables, we measured individuals' sense of autonomy and capacity to engage with or make decisions about AI use, especially in high-stakes healthcare contexts. Perceived usefulness, informed by the Technology Acceptance Model, refers to the belief that AI integration may improve clinical outcomes. These constructs help explain our participants' willingness to accept AI applications across the progressively complex scenarios described.

*Positive expectations* were measured by asking doctors to rate five items on a 7-point scale (“impossible to happen” to “certain to happen”) about what they expect will happen when AI is increasingly used in gastroenterological diagnosis. Questions included: “There will be fewer medical errors” and “AI will improve diagnostic efficiency”. Unidimensionality of the underlying construct was assured via Confirmatory Factor Analysis (CFA) (McDonald's *ω*=0.88, M = 5.75, SD = 1.02; *p*-value = 0.039, CFI = 0.978, RMSEA = 0.0663, SRMR = 0.0247).

*Negative expectations* were measured on the abovementioned 7-point scale. Questions included: “AI will eventually replace pathologists”, and “AI will hinder clinician-patient relationship”. Again, the unidimensionality of the underlying construct was assured via CFA (McDonald's *ω*=0.76, M = 2.96, SD = 1.20; *p*-value = 0.79, CFI = 1.000, RMSEA = 0.000, SRMR = 0.0167).

*Perceived control* refers to the extent of control clinicians perceived regarding own decisions with AI introduction, measured with four items on a 7-point Disagree – Agree scale. Using CFA, unidimensionality of construct was satisfactory (*p*-value = 0.197, CFI = 0.995, RMSEA = 0.044, SRMR = 0.011); composite reliability measured by McDonald's *ω* was good (0.88, M = 3.43, SD = 1.42).

*Attitude towards AI* was an averaged score composed out of six pairs of evaluations regarding how it feels for the respondent that AI will be used in patient care. Evaluations were made on pairs such as bad/good, frightening/reassuring, worthless/valuable, and unpleasant/pleasant, on a 7-point scale. Unidimensionality of the attitude was assured via CFA (McDonald's *ω*=0.94, M = 5.75, SD = 1.02; *p*-value = 0.058, CFI = 0.979, RMSEA = 0.0593, SRMR = 0.0218).

Participants' level of *trust* was measured with six items, two items per scenario. In each scenario, clinicians were asked to imagine a conversation with a colleague regarding an application of AI in gastroenterology, specifically related to colonoscopy and managing colorectal polyps. This variable was assessed more in-depth in our pilot study, thus was not a key variable for analysis in this study (9). Participants were asked two questions: (1) “Do you fully believe the colleague, or will you harbor doubts?”, and (2) “Do you believe that machine learning algorithm can, in some cases (as in the scenario), perform better than human beings?” For the first item, harboring doubts refers to clinicians' doubts related to the AI application in each scenario. Items were rated on a 7-point Likert scale. A mean score from the six items was calculated (McDonald's *ω*=0.86, M = 5.07, SD = 1.05).

For *acceptance,* an overall mean score of six items was calculated based on responses to two questions in each of the three scenarios. Specifically, whether they would accept the presented method in the scenario and whether they would be ready to try the method themselves (McDonald's *ω*=0.91, M = 5.33, SD = 1.09).

*Acceptance per scenario:* To test changes in acceptance levels, we measured acceptance for each scenario composing of an average of two items: scenario 1 acceptance (McDonald's *ω*=0.87, M = 5.39, SD = 1.19), scenario 2 acceptance (McDonald's *ω*=0.88, M = 5.43, SD = 1.22), and scenario 3 acceptance (McDonald's *ω*=0.85, M = 5.17, SD = 1.25).

To determine the *amount of experience and level of seniority* participants had in their specialty, respondents were asked: “How many years (in total full-time practice) have you have been practicing in your specialty?” Participants were given four options: <5 years, 5–10 years, 11–20 years, and >20 years.

The measure for *perceived risk* of AI use in gastroenterology practice included three items. Participants rated the following statement: “I expect major risks involved with the AI” for each of the three scenarios on a 7-point Likert scale. A composite score for risk was calculated. Then, a mean risk score (minimum = 1, maximum = 7) across scenarios was calculated. The variable was subsequently dichotomized based on the median, splitting respondents into two equal sized groups. Participants who scored ≤4 were considered to have “low” levels of perceived risk, and those who scored ≥5 were categorized to have “high” levels of perceived risk. The omega-hierarchical coefficient for interval-level item responses was 0.86 (M = 3.97, SD = 1.43), signifying high reliability.

### 2.3 Analysis

Participants' characteristics, including socio-demographic, job-specific, and AI-related details, were summarized using descriptive statistics. Independent T-tests were employed to analyze differences between more and less experienced clinicians regarding their risk perception, and expectations regarding AI applications in gastroenterology. Based on the results, participants were divided into four groups, namely those who have (i) low experience and low risk perception (LELR), (ii) low experience and high risk perception (LEHR), (iii) high experience and low risk perception (HELR), and (iv) high experience and high risk perception (HEHR). A chi-square test of independence and regression analysis was performed to examine whether these four groups were independent. Next, an ANCOVA model was constructed to test whether the four groups differ regarding their overall acceptance of AI. Finally, a repeated-measures ANOVA was performed to evaluate whether the differences were stable over three scenarios. All data analysis was conducted in SPSS version 29.0 (19).

## 3 Results

### 3.1 Participant descriptives

Numbers and percentages reported below are based on the total number of participants (*n* = 319) who responded to the question, with missing values excluded. The average age of the participants was 43.76 years (SD = 8.70 years). Most participants specialized in gastroenterology (*n* = 278, 87.10%), came from public institutions (*n* = 258, 80.09%), and worked in departments with more than 10 staff members (*n* = 210, 68.40%). There were slightly more male participants (*n* = 168, 52.66%). 145 participants (45.40%) had 10 years or less of experience in gastroenterology. More participants had low-risk perceptions of Artificial Intelligence (AI) in healthcare (*n* = 181, 56.74%).

### 3.2 Comparing differences between the experience of clinicians

**Figure 1:**
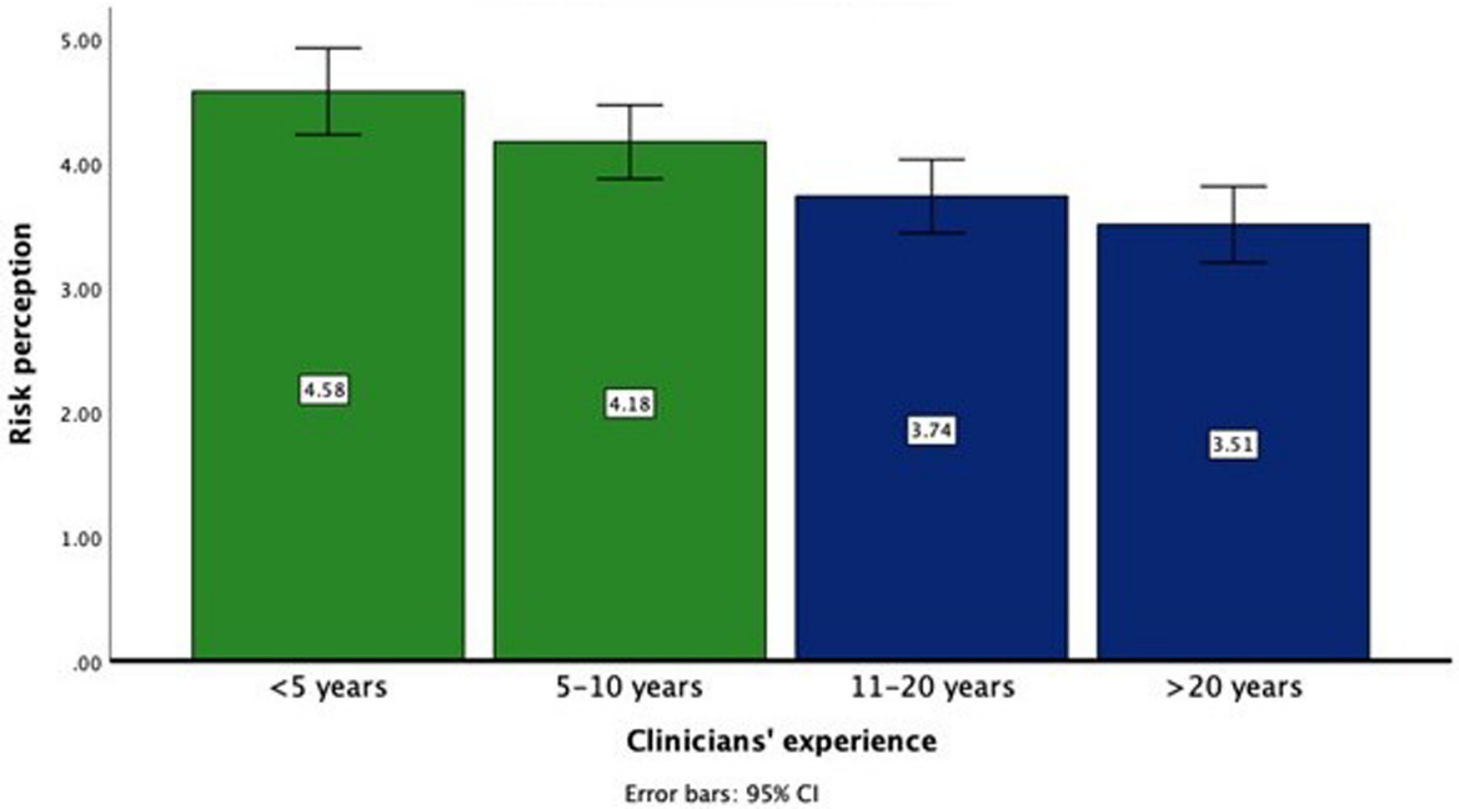
Risk perception towards AI applications in Gastroenterology by levels of clinicians’ experience.

Regarding *risk perception* across the four levels of seniority, there was a consistent decrease in risk aversion: less experienced clinicians were more risk-averse compared to the experienced gastroenterologists (Figure 1). The risk perception scores for the groups were as follows: the least experienced clinicians (M = 4.58, SD = 1.27), junior (M = 4.18, SD = 1.45), mid-level (M = 3.74, SD = 1.47), and senior (M = 3.51, SD = 1.28). The ANOVA indicated a significant effect of experience level on AI-related risk perceptions, *F*(3, 308) = 8.288, *p* < 0.001, with a medium effect size (*η*^2^ = 0.075). *post-hoc* comparisons using Tukey's HSD indicated that senior clinicians reported significantly lower risk perception scores than junior (*p* < 0.05) or least experienced clinicians (*p* < 0.001), and mid-level clinicians reported significantly lower risk perceptions compared to the least experienced gastroenterologists (*p* < 0.01). The differences between senior and mid-level clinicians, and between mid-level and junior clinicians was not statistically significant.

For the following analysis, the four levels of seniority were dichotomized based on a Median score: less experience (<10 years) and higher experience (>10 years).

Higher experienced clinicians showed a more positive attitude towards AI (*M* = 5.76, SD = 1.18) compared to less experienced clinicians [*M* = 5.40, SD = 1.29, *t*(271) = 2.402, *p* = 0.017]. They were, generally less perceive risks to be lower (*M* = 3.63, SD = 1.38) compared to less experienced colleagues [*M* = 4.35, SD = 1.39, *t*(307) = 4.536, *p* < 0.001]. There were no significant differences found regarding positive or negative expectations towards AI among clinicians with different experience levels. Results are summarized in Table 1.

**Table 1:** Comparing attitude, risk perception, and positive/negative expectations between high-experienced (>10 years) and low-experienced (<10 years) clinicians regarding AI applications.

	Level of Experience	M(SD)	t/df	*p*-value/Cohen's d
Attitude towards AI	High	5.76 (1.29)	2.40/271	0.017/0.29
	Low	5.740 (1.18)		
Risk perception of AI	High	3.63 (1.38)	−4.54/307	<0.001/0.52
	Low	4.35 (1.39)		
Positive expectations towards AI	High	5.83 (1.00)	1.68/307	0.094
	Low	5.64 (1.04)		
Negative expectations towards AI	High	2.94 (1.17)	−0.51/303	0.607
	Low	3.01 (1.26)		
Belief that quality of care will increase	High	5.13 (1.08)	1.49/307	0.138
	Low	4.95 (1.03)		
AI can decrease doctor's control over the care process	High	3.39 (1.51)	−0.42/306	0.677
	Low	3.46 (1.34)		
Being familiar with AI	High	2.59 (0.79)	2.23/307	0.026/0.25
	Low	2.39 (0.76)		

10 participants did not report their experience levels and their data were not considered for this part of the analysis.

### 3.3 Participant categorization

**Figure 2:**
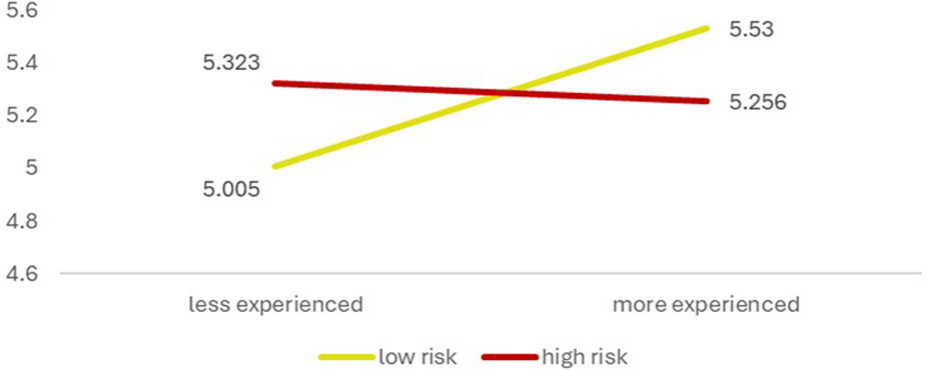
Categorization of clinicians based on level of experience and risk perception.

Using levels of experience (low vs. high-experienced) and risk perception (low vs. high-risk), participants are categorized into four groups (Figure 2).

ANOVA analysis revealed a significant effect of experience, risk perception, and the covariate (attitude) together on AI acceptance, *F*(4, 268) = 32.079, *p* < 0.001, partial *η*^2^ = 0.324. The covariate, clinicians' attitude towards AI, was found to have a significant impact on AI acceptance, *F*(1, 268) = 106.845, *p* < 0.001, partial *η*^2^ = 0.285. This suggests that clinicians with a more favorable attitude towards AI tend to have higher acceptance of AI applications. The level of experience was also significant, *F*(1, 268) = 4.136, *p* = 0.043, partial *η*^2^ = 0.015, implying that clinicians' experience level influences their acceptance of AI. More experienced clinicians showing higher levels of acceptance compared to their less experienced counterparts. However, the main effect of risk perception was not significant, suggesting that clinicians' risk perceptions regarding AI, independent from their seniority level, do not significantly impact their acceptance.

**Figure 3:**
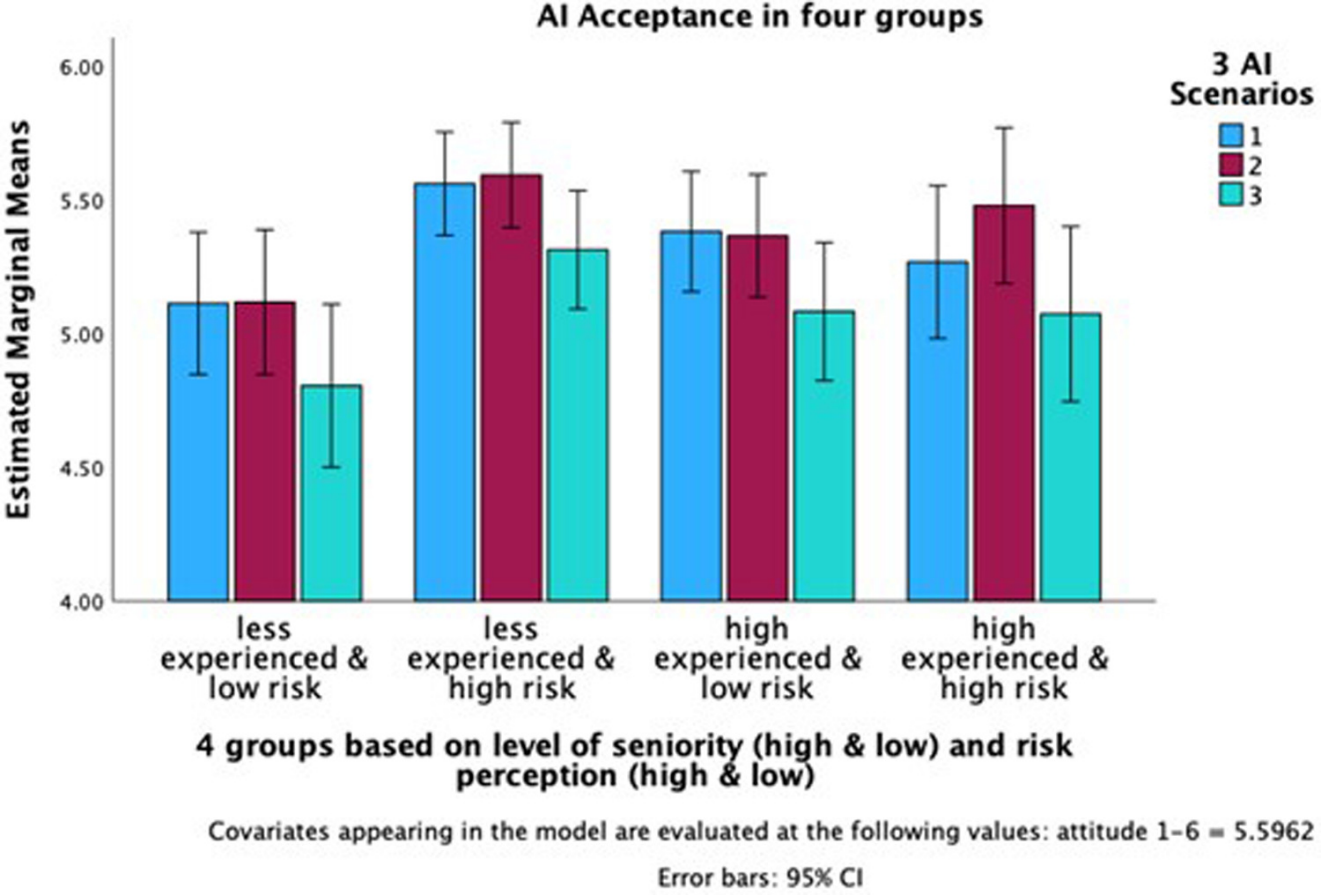
Interaction effect of clinicians’ experience and risk perception on AI acceptance. *The *y*-axis indicates AI acceptance and starts at 4.00 to underscore the group differences.

The interaction effect between experience level and risk perception was significant, *F*(1, 268) = 6.991, *p* = 0.009, partial *η*^2^ = 0.025, indicating that the effect of experience on AI acceptance varies according to the clinicians' risk perceptions (Figure 3). Specifically, the combination of having high experience with low or high-risk perceptions influences AI acceptance differently compared to other combinations of experience and risk perception. In other words, the effect of experience on AI acceptance is not uniform across all clinicians. It depends on the perceived risks associated with it. Highly experienced clinicians may exhibit higher acceptance when they perceive the risks as low, whereas acceptance decreases if they perceive the risks as high. Less experienced clinicians indicate a different pattern. Their acceptance of AI is high when they exhibit a high level of being risk averse, and acceptance is low when their risk perception is equally low.

The results indicate that while clinicians’ experience level and their attitude towards AI significantly impact acceptance of AI in their clinical practice, risk perception alone does not. However, the interaction between experience and risk perception suggests that the relationship between experience and acceptance is moderated by clinicians' risk perceptions of AI.

**Figure 4:**
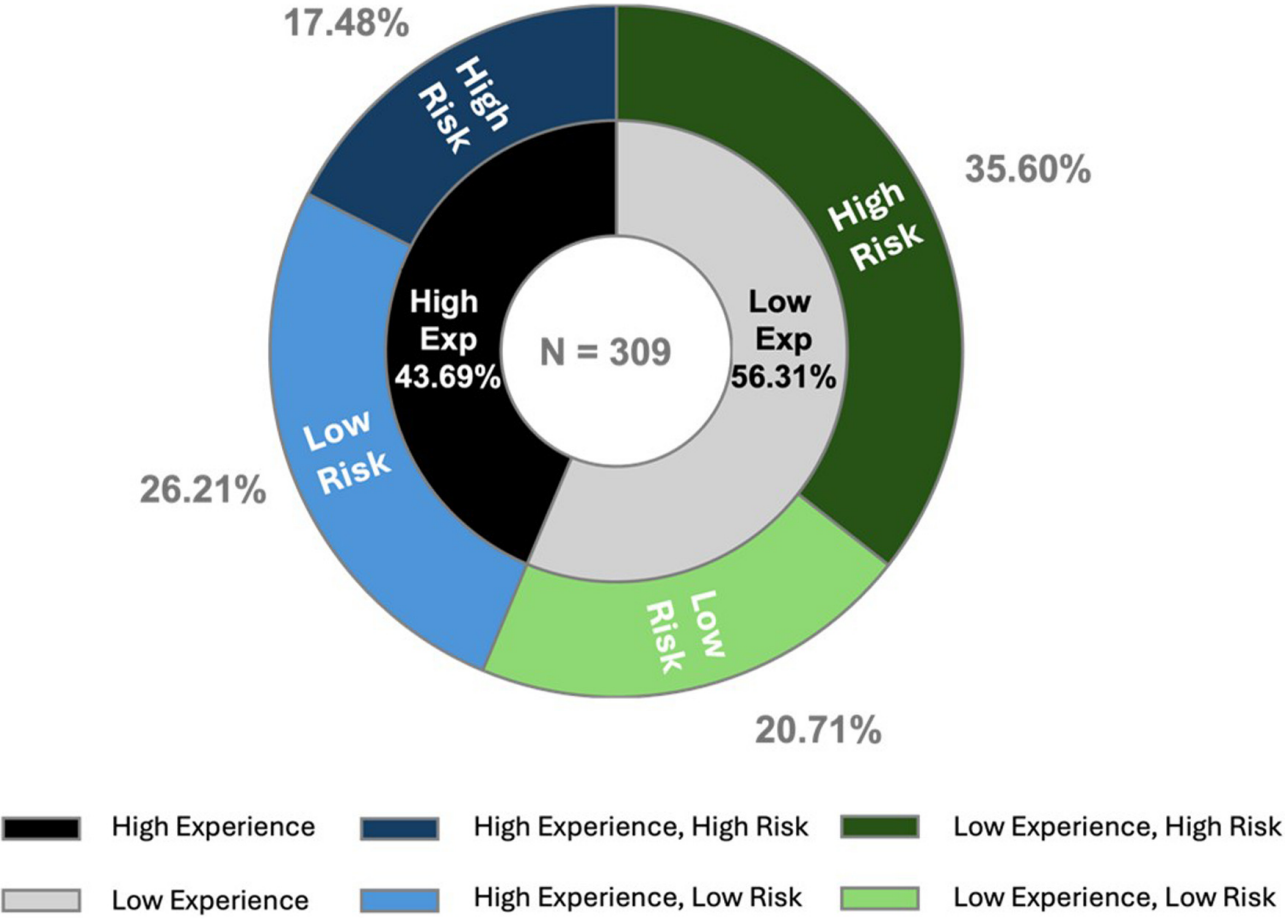
Ai acceptance in all three scenarios for all four groups. *The *y*-axis indicates AI Acceptance and starts at 4.00 to underscore the group differences.

Finally, we conducted a repeated-measures ANOVA to test the interaction effect of experience level and risk perception differs between the scenarios. The effect of the scenarios on acceptance was not significant. *post-hoc* pairwise comparisons (with Tukey adjustment showed no significant difference between the acceptance in the first scenario and the second scenario the second and third scenario, and the first and third scenario. Results indicate that the interaction effect of level of experience/risk perception groups on the acceptance in all three scenarios was significant (*p* < 0.001) with AI acceptance in the first scenario, *F*(3, 305) = 8.279; for the second scenario *F*(3, 305) = 6.252, and the third scenario *F*(3, 305) = 5.909. Thus, the interaction of clinicians' experience and their risk perception is independent of the AI application complexity. acceptance among the four groups diminished, though they remained significant (Figure 4).

## 4 Discussion

This study aimed to understand how the risk perception and clinician experience interact to influence clinicians’ Artificial Intelligence (AI) acceptance. Results suggest that experienced clinicians are generally more likely to accept AI applications in gastroenterology at different levels of complexity than their junior colleagues. Among senior and experienced gastroenterologists, those with lower risk perceptions are more willing to accept AI. Therefore, experience is the primary determining factor and perception of risk takes a secondary role in determining acceptance of AI in Gastroenterology.

AI has been increasingly used in various medical specialties, from diagnosis, risk prediction, suggestions of treatment, and surveillance of diseases. Because of the maturity of AI technology in image interpretation, it is more widely adopted. AI tools are useful in interpreting radiology, pathology, and endoscopy images in diagnosing benign and malignant conditions such as inflammatory bowel disease, gastrointestinal cancers, and liver diseases. The use of AI colonoscopy in detecting and treating colorectal polyps is the most studied and debated condition among GI and liver conditions. How much clinicians trust and accept the use of AI-assisted colonoscopy determines whether its implementation will be successful.

Our previous pilot study indicated that young gastroenterologists are less likely to trust and accept AI-assisted colonoscopy in the diagnosis and treatment (9). This is somewhat out of what we expected. Younger generations, raised in the era of the internet, smartphones, and AI, may be more likely to accept technology, including in medical decisions and interventions. In that study, we found that the only factor that significantly predicted trust and acceptance was years of experience in endoscopy. The findings of this study echo this previous finding, that experienced endoscopists expressed a higher acceptance of using AI technology than young endoscopists. However, for the less experienced physicians this pattern does not apply. Their acceptance of the integration of AI is not as dependent on their risk perception. Junior clinicians, in general, have low acceptance of using AI irrespective of their perceived risk level. This may reflect a belief that while AI offers valuable support or may be able to compensate for potential gaps, having a lower level of experience may lead to views that can pose a challenge for younger clinicians. Even among those who perceive AI as relatively low-risk, acceptance remains limited, suggesting that other factors may be shaping their reluctance. This could include a lack of confidence in interpreting AI outputs or concerns that AI may undermine their autonomy as clinicians. Other studies that investigated whether higher levels of risk perception predict lower acceptance of AI yielded similar results. One study found that risk perception mediates the relationship between perceptions of AI and the intention to use it, suggesting that clinicians with higher perceived risks are less inclined to utilize AI (20). A review identified that AI acceptance is influenced by specific risks, such as the risk of medical errors and bias (21). Another review identified that both human and technology-related factors affect overall acceptance of AI in healthcare practices (22). Thus, we speculate that senior gastroenterologists with ample experience in endoscopy know that they can exercise discretion based on professional knowledge and experience and AI merely acts as an assistive tool. These findings align with the Technology Acceptance Model and the Theory of Planned Behavior, which both identify user attitudes as a primary predictor of technology adoption. This emphasizes the findings that clinicians' acceptance of AI is most strongly influenced by their underlying evaluations of its usefulness and impact. The final decision rests on their experience and the knowledge of patients which may not be reflected on the clinical notes or endoscopic image. Among younger gastroenterologists with limited experience, there may be hesitancy utilizing AI, particularly when making critical treatment decisions. Hence, their acceptance of AI-assisted colonoscopy may be more constrained.

The moderation effect of clinical experience and risk perception seems to be independent of various levels of complexity of AI applications as they were presented in our scenarios. More experienced clinicians perceive themselves as being capable and willing to utilize AI across scenarios. Level of experience, which is related to seniority, appears to play a significant role in AI acceptance, less experienced gastroenterologists may perceive that AI presents an input-output imbalance. In other words, the perceived amount of time and cognitive effort required to understand and apply AI tools are seen as disproportionate to the amount of clinical value or benefit it provides to a clinician. Specifically, less experienced clinicians who consider the risk of AI low assign lower value to AI. They may believe that additional effort, such as learning use and interpret the results from AI, yields minimal benefit. This perception likely contributes to their lower acceptance of AI. Additionally, they might view AI as a potential threat to their professional roles. We could not test this variable directly; this should be studied in future research. In contrast, senior gastroenterologists who consider the risk of AI to be low, as they may assume that their expertise will allow them to manage it effectively. They do not see technological advancements as a threat to their profession; rather, they believe their skills will remain indispensable. Although the main and interaction effects were statistically significant, effect sizes were small to moderate. This, combined with a modest sample size, may have limited the detection of more subtle effects. Still, the findings offer robust preliminary insights and a strong basis for future work.

The application of AI in healthcare is extremely varied. This study focused on AI-assisted colonoscopy in the diagnosis and management of colorectal polyps. With the rapid development in generative pre-trained transformer (GPT) architecture, new opportunities have emerged for AI to assist in managing gastrointestinal conditions, including patient communication, therapy recommendations, and post-colonoscopy counseling. Large Language Model (LLM) GPTs (like ChatGPT) have been shown to provide satisfactory responses to patient queries related to colonoscopy (23). However, another study noted that while ChatGPT can answer gastroenterology-related questions, its responses often fall below the professional level due to a lack of up-to-date information (24). Technological advancements will likely bridge this gap in the future. LLMs have been found to assist healthcare providers in making informed decisions and improving adherence to post-colonoscopy surveillance guidelines (25). Furthermore, the findings from this study focused on the specific applications of AI in the domain of gastroenterology. It is imperative for similar studies to be conducted in different domains of healthcare, such as psychiatry, where different forms of AI may be integrated into healthcare, to gain a more in-depth understanding of the impact of AI use in these domains. Regardless, the findings of this study indicate that, in general, medical practitioners who are more experienced are more willing to utilize AI in their general practice, likely due to their own confidence in being able to navigate any scenarios they encounter. These findings suggest several practical implications, including leveraging senior clinicians as champions for AI implementation, to assist in designing or delivering training programs for junior clinicians to improve familiarity and perceived usefulness, and addressing risk-related concerns early to build trust across experience levels. As technology continues to evolve, early engagement and training of healthcare providers will be critical for the successful implementation of AI in healthcare.

## 5 Conclusions

Our findings revealed that the combination of clinician experience and risk perception interactively influence the acceptance of Artificial Intelligence (AI). More experienced clinicians were more likely to embrace AI compared to their junior counterparts, particularly when they perceived the risk as being low. Future studies should explore additional factors that can enhance clinician's trust and increase their acceptance of AI technologies in providing better clinical assistance and care to their patients. Understanding these dynamics will be crucial not only for successful AI integration but also for shaping future models of collaborative, human–AI decision-making in medicine.

## Data Availability

The raw data supporting the conclusions of this article will be made available by the authors, without undue reservation.

## References

[1] Schwalbe N Wahl B. Artificial intelligence and the future of global health. Lancet. (2020) 395:1579–86. 10.1016/S0140-6736(20)30226-9 32416782 PMC7255280

[2] Rajpurkar P Chen E Banerjee O Topol EJ. AI In health and medicine. Nat Med. (2022) 28:31–8. 10.1038/s41591-021-01614-0 35058619

[3] Oren O Gersh BJ Bhatt DL. Artificial intelligence in medical imaging: switching from radiographic pathological data to clinically meaningful endpoints. Lancet Digit Health. (2020) 2:e486–e8. 10.1016/S2589-7500(20)30160-6 33328116

[4] Kröner PT Engels MM Glicksberg BS Johnson KW Mzaik O van Hooft JE Artificial intelligence in gastroenterology: a state-of-the-art review. World J Gastroenterol. (2021) 27:6794–824. 10.3748/wjg.v27.i40.6794 34790008 PMC8567482

[5] Sung JJ Poon NC. Artificial intelligence in gastroenterology: where are we heading? Front Med. (2020) 14:511–7. 10.1007/s11684-020-0742-4 32458189

[6] Ishikawa T Yamao K Mizutani Y Iida T Kawashima H. Cutting edge of endoscopic ultrasound-guided fine-needle aspiration for solid pancreatic lesions. J Med Ultrason. (2024) 51:209–17. 10.1007/s10396-023-01375-y PMC11098899 37914883

[7] Solanki SL Pandrowala S Nayak A Bhandare M Ambulkar RP Shrikhande SV. Artificial intelligence in perioperative management of major gastrointestinal surgeries. World J Gastroenterol. (2021) 27:2758–70. 10.3748/wjg.v27.i21.2758 34135552 PMC8173379

[8] Leggett CL Parasa S Repici A Berzin TM Gross SA Sharma P. Physician perceptions on the current and future impact of artificial intelligence to the field of gastroenterology. Gastrointest Endosc. (2024) 99:483–9.e2. 10.1016/j.gie.2023.11.053 38416097

[9] Goh WW Chia KY Cheung MF Kee KM Lwin MO Schulz PJ Risk perception, acceptance, and trust of using AI in gastroenterology practice in the Asia-Pacific region: web-based survey study. JMIR AI. (2024) 3:e50525. 10.2196/50525 38875591 PMC11041476

[10] Huang Z George MM Tan Y-R Natarajan K Devasagayam E Tay E Are physicians ready for precision antibiotic prescribing? A qualitative analysis of the acceptance of artificial intelligence-enabled clinical decision support systems in India and Singapore. J Glob Antimicrob Resist. (2023) 35:76–85. 10.1016/j.jgar.2023.08.016 37640155 PMC10684720

[11] Wang W Gao G Agarwal R. Friend or foe? Teaming between artificial intelligence and workers with variation in experience. Manage Sci. (2024) 70:5753–75. 10.1287/mnsc.2021.00588

[12] Shinners L Aggar C Grace S Smith S. Exploring healthcare professionals’ understanding and experiences of artificial intelligence technology use in the delivery of healthcare: an integrative review. Health Informatics J. (2020) 26:1225–36. 10.1177/1460458219874641 31566454

[13] Ajzen I. The theory of planned behaviour: reactions and reflections. Psychol Health. (2011) 26:1113–27. 10.1080/08870446.2011.613995 21929476

[14] Ajzen I. The theory of planned behavior. Organ Behav Hum Decis Process. (1991) 50:179–211. 10.1016/0749-5978(91)90020-T

[15] Davis FD. User acceptance of information systems: the technology acceptance model (TAM) (doctoral dissertation). University of Michigan, School of Business Administration, Division of Research, Ann Arbor, MI, United States. (1987). Available online at: https://deepblue.lib.umich.edu/handle/2027.42/35547

[16] Schulz PJ Lwin MO Kee KM Goh WWB Lam TYT Sung JJY. Modeling the influence of attitudes, trust, and beliefs on endoscopists’ acceptance of artificial intelligence applications in medical practice. Front Public Health. (2023) 11:1301563. 10.3389/fpubh.2023.1301563 38089040 PMC10715310

[17] Hah H Goldin DS. How clinicians perceive artificial intelligence–assisted technologies in diagnostic decision making Mixed Methods Approach. J Med Internet Res. (2021) 23:e33540. 10.2196/33540 34924356 PMC8726017

[18] Kader R Baggaley RF Hussein M Ahmad OF Patel N Corbett G Survey on the perceptions of UK gastroenterologists and endoscopists to artificial intelligence. Front Gastroenterol. (2022) 13:423–9. 10.1136/flgastro-2021-101994 PMC9380773 36046492

[19] SPSS I. IBM SPSS Statistics for Windows. Armonk, New York, USA: IBM SPSS (2013) 2:119

[20] Choudhury A. Factors influencing clinicians’ willingness to use an AI-based clinical decision support system. Front Digit Health. (2022) 4:920662. 10.3389/fdgth.2022.920662 36339516 PMC9628998

[21] Chew HSJ Achananuparp P. Perceptions and needs of artificial intelligence in health care to increase adoption Scoping Review. J Med Internet Res. (2022) 24:e32939. 10.2196/32939 35029538 PMC8800095

[22] Shevtsova D Ahmed A Boot IWA Sanges C Hudecek M Jacobs JJL Trust in and acceptance of artificial intelligence applications in medicine: mixed methods study. JMIR Hum Factors. (2024) 11:e47031. 10.2196/47031 38231544 PMC10831593

[23] Lee TC Staller K Botoman V Pathipati MP Varma S Kuo B. ChatGPT answers common patient questions about colonoscopy. Gastroenterology. (2023) 165:509–11.e7. 10.1053/j.gastro.2023.04.033 37150470

[24] Suchman K Garg S Trindade AJ. Chat generative pretrained transformer fails the multiple-choice American college of gastroenterology self-assessment test. Am J Gastroenterol. (2023) 118:2280–2. 10.14309/ajg.0000000000002320 37212584

[25] Gorelik Y Ghersin I Maza I Klein A. Harnessing language models for streamlined postcolonoscopy patient management: a novel approach. Gastrointest Endosc. (2023) 98:639–41.e4. 10.1016/j.gie.2023.06.025 37385548

